# The role of redox system in metastasis formation

**DOI:** 10.1007/s10456-021-09779-5

**Published:** 2021-04-28

**Authors:** Chiara Cencioni, Valentina Comunanza, Emanuele Middonti, Edoardo Vallariello, Federico Bussolino

**Affiliations:** 1 Institute for Systems Analysis and Computer Science “A. Ruberti”, National Research Council (IASI-CNR), 00185 Rome, Italy; 2 Department of Oncology, University of Torino, 10043 Orbassano, Italy; 3 Candiolo Cancer Institute – IRCCS-FPO, 10063 Candiolo, Italy; 4 Strada Provinciale di Piobesi 142, Km 3.95, 10060 Candiolo, Italy

**Keywords:** Neutrophils, Endothelial cells, Metastatic cancer cells, Platelets

## Abstract

The metastatic cancer disease represents the real and urgent clinical need in oncology. Therefore, an understanding of the complex molecular mechanisms sustaining the metastatic cascade is critical to advance cancer therapies. Recent studies highlight how redox signaling influences the behavior of metastatic cancer cells, contributes to their travel in bloodstream from the primary tumor to the distant organs and conditions the progression of the micrometastases or their dormant state. Radical oxygen species not only regulate intracellular processes but participate to paracrine circuits by diffusion to nearby cells, thus assuming unpredicted roles in the communication between metastatic cancer cells, blood circulating cells, and stroma cells at site of colonization. Here, we review recent insights in the role of radical oxygen species in the metastasis formation with a special focus on extravasation at metastatic sites.

**Supplementary Information:**

The online version contains supplementary material available at 10.1007/s10456-021-09779-5.

## Introduction

Metastasis spreading from the primary lesion to secondary sites causes over 90% of human cancer deaths [1]. Fortunately, it is evident that the number of patients living with a controlled and dormant metastatic disease is increasing in the last twenty years [2].

Metastasis diffusion is a multiple step process with different bottlenecks, which renders the journey of cancer cells in bloodstream highly demanding with few chances to get to the final destination. Although, primary tumors can discard millions of cells into the capillaries every day, a metastatic event occurs rarely. The cancer population accountable for the metastatic process is defined metastatic cancer cell (MCC), the protagonist of cancer progression. The first step is the intravasation of MCCs into the tumor vascular bed. Then, MCCs move into the blood as a single cell or clusters, possibly surrounded by platelets or polymorphonuclear cells (PMNs). Patterns of MCCs in the bloodstream and features of the vasculature in each organ influence the efficacy of the metastatic process. Furthermore, there are increasing evidences suggesting a role of primary tumor secretome in dictating the selection of metastatic sites. For example, primary tumors release angiocrine molecules or chemokines able to directly or indirectly address bone marrow-derived hematopoietic progenitors at site of metastatic spreading to induce an immune-suppressive environment [3]. Primary tumor cells might also release exosomes, which fuse with resident cells at their predicted destination and transfer their cargo to prepare the pre-metastatic niche [4]. Alternatively, in the presence of shear forces MCCs discharge cytosolic microparticles, which are ingested by different myeloid cell types in progressive and temporally differentiated waves to prepare the features of the metastasis homing. According to the myeloid cell types, the MCC cargos might address their functions towards a pro- or anti-metastatic effect [5].

**Fig. 1:**
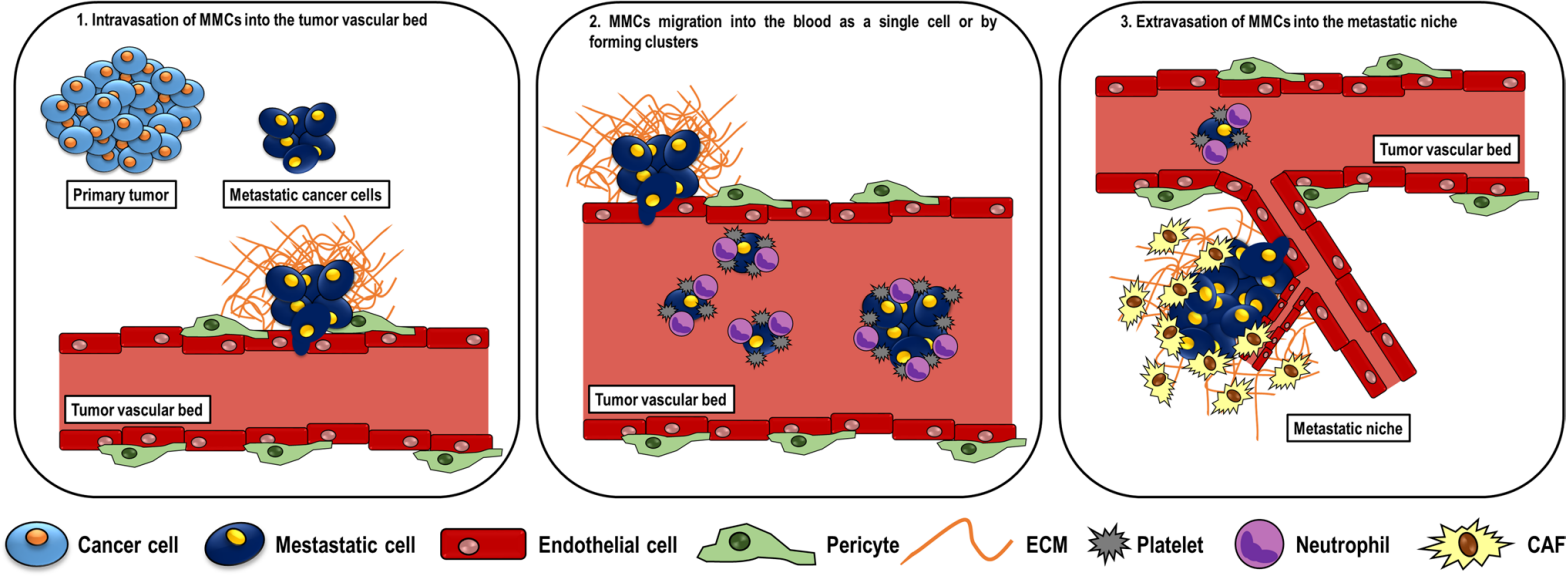
Steps of the metastatic process. Schematic representation of the three steps of metastatic process focusing on the role of Metastatic Cancer Cells, responsible for cancer progression

When MCCs stop at the capillary surface of targeted tissues, they extravasate into the parenchyma and settle within an area near the capillaries termed “metastatic niche [6]”, where they remain in dormant state or start to invade surrounding tissues (Fig. 1).

This scenario is well supported by a huge amounts of in vitro and in vivo data [1, 7]. Nevertheless, the molecular and biological bases underpinning metastatic diffusion remain barely investigated and understood. For this reason, many questions are still unsolved limiting the design of successful therapeutic approaches able to counteract the metastatic disease. Which are the mechanisms sustaining the organ tropism from the primary tumor to the site of metastatic seeding? Which are the features of cells moving out the primary tumor with the capability to colonize distant organs? Which are the minimal requirements sustaining survival and expansion of a metastatic clone in a distant and often hostile organ?

This review will focus on the role of redox state and radical oxygen species (ROS) in influencing the success rate of metastatic process analyzing the extravasation and the settlement of MCCs in distant organs [6, 8]. Low and physiologic concentrations of ROS represent a rapid and versatile tool for fine-tuning many intracellular processes [9], and might dynamically regulate oncogenesis. A paradigmatic example of this concept has been recently provided in pancreatic adenocarcinoma models by analyzing the role of TP53-induced glycolysis and apoptosis regulator (TIGAR), a bisphosphatase that activates the oxidative pentose phosphate pathway to generate antioxidant nicotinamide adenine dinucleotide phosphate (NADPH). In this study high levels of TIGAR first promote pancreatic cancer initiation by limiting ROS, thereafter low levels of TIGAR promote the metastatic capacity of pancreatic cancer cells by enhancing ROS [10].

Furthermore, recent observations extended their regulatory roles from intracellular mechanisms to signal circuits occurring between single cells. ROS might be generated within cells before being released in a paracrine manner diffusing to nearby target cells, or ROS-generating systems might be delivered through exosomes to affect the behavior of neighboring cells [11].

## Sources of radical species

Endogenous free radicals, both ROS and reactive nitrogen species (RNS), are produced in several sub-cellular organelles (mitochondria, peroxisomes, endoplasmic reticulum) and according to their concentrations and half-time exert both signaling and oxidative damage on lipids, nucleic acids and proteins.

**Fig. 2:**
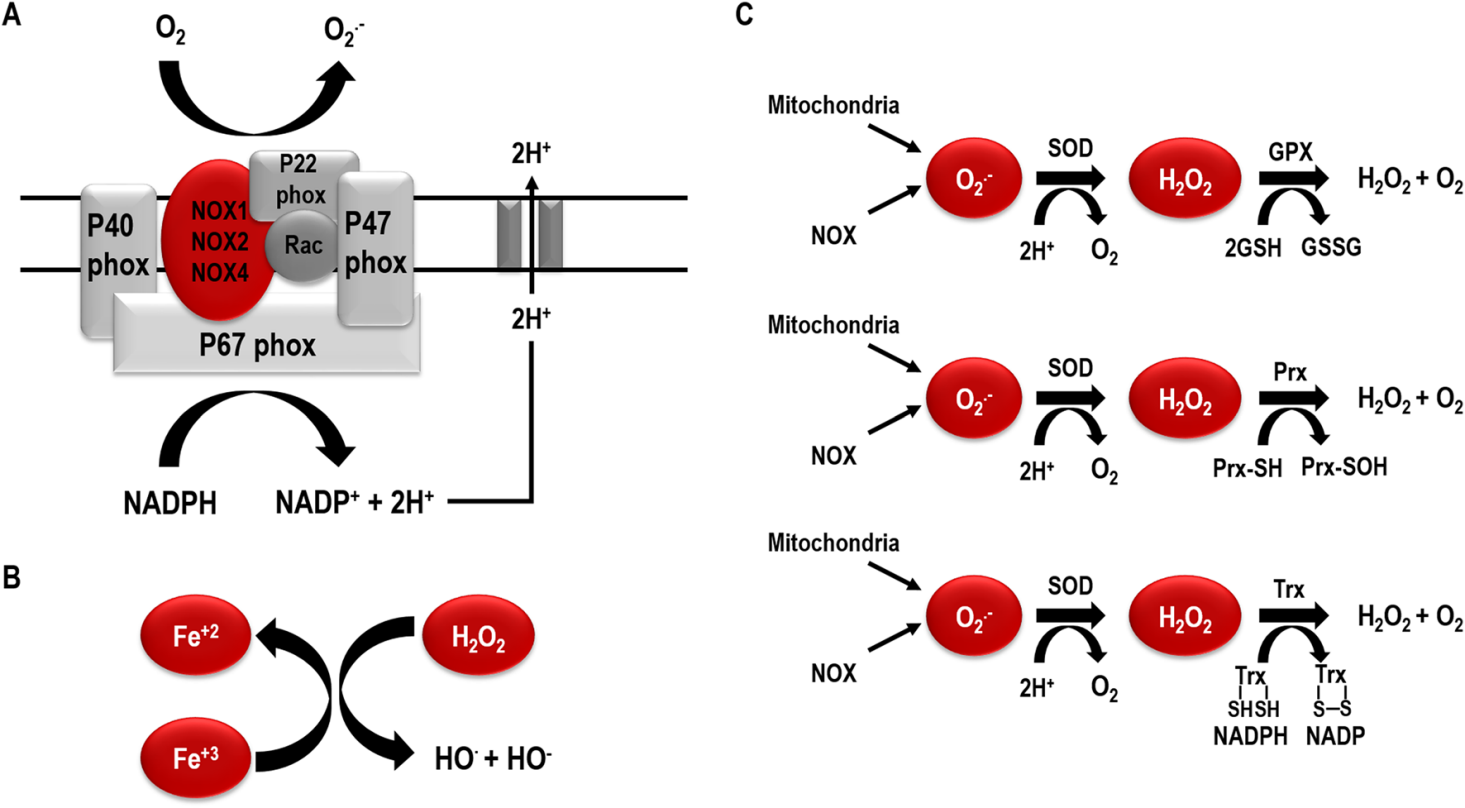
Source of oxidative stress. Schematic representation of ROS production and scavenging. **a** Superoxide is mainly generated from the oxidation of NADPH by NOXs or mitochondria through electron chain complexes I and III. ROS might result from the enzymatic activity of membrane-bound NOX. NOXs consume NADPH to generate O_2_^.−^ and then H_2_O_2_. **b** Fenton reaction: catalytic reaction converting hydrogen peroxide in the presence of ferrous ions into a highly toxic hydroxyl free radical. **c** The scavenging system involving the catalase or GPX coupled with GSH oxidation, Prx and Trx

The first step of cellular production of ROS is the reduction of O_2_ to superoxide ion (O_2_^•−^). Almost 95% of O2^•−^ is unspecifically produced along the electron transfer through the complexes of the mitochondrial electron transport chain. The residual production is catalyzed by NADPH oxidase (NOX) family, which transfers electrons from NADPH to O_2_. Superoxide is then detoxified to generate other ROS molecules, which include hydroxyl radical, aloxyl, peroxyl, singlet oxygen and non-radical H_2_O_2_, which is the primary mediator of ROS-driven cellular signaling (Fig. 2a). In particular in mitochondria, where Fe^++^ can be released by damaged cytochromes upon O_2_^•−^ generation, H_2_O_2_ converts to hydroxyl radical through Fenton’s reaction (Fig. 2b) [9].

The most relevant detoxifying enzyme is the superoxide-dismutase (SOD), which converts superoxide into H_2_O_2_. According to the cell status and the subcellular localization, H_2_O_2_ is differently processed. It might be converted into water by the reductase activity of peroxiredoxin (Prx), catalase, thioredoxin (Trx) reductase and glutathione (GSH) peroxidase-GSH reductase system (Fig. 2c) [9, 12].

ROS metabolism is strictly connected with RNS and in particular with nitric oxide (NO), which is synthesized by various isoforms of nitric oxide synthase (NOS). NO can further react with superoxide to form peroxynitrite ONOO^−^, which mediates the nitrosylation or the oxidation of amino acid residues such as tyrosine, cysteine, methionine, and tryptophan.

Evolution has developed highly organized systems to quench the dangerous effect of the excessive ROS production or fine-tune their physiologic role [12]. These antioxidant pathways include plasmatic small molecules (bilirubin, urate, ascorbate, vitamin E) and proteins (albumin, ceruloplasmin, ferritin) that sequester metallic ions (Fe^++^, Cu^+^) undergoing oxidation and the enzymatic reductive systems [12]. Furthermore, the cell capability to control ROS effects is further orchestrated by the Nuclear factor erythroid 2-related factor-2 (NRF-2), which regulates the transcription of genes involved in the biosynthesis of NADPH, GSH and in the Prx-Trx pathway. Under redox homeostasis, cytosolic NRF-2 binds Kelk-like ECH-associated protein 1 and undergoes degradation. When ROS are abundant, the formation of this complex is limited and NRF-2 translocates to the nucleus, where it promotes an antioxidant transcriptional program [13].

## Features of the metastatic cancer cells

There is growing evidence that tumorigenesis is fueled by a minute population of cells endowed with unique self-renewal potential and arising when transit-amplifying cells with mutant genomes differentiate and enter the stem cell state. These cells are operationally classified as initiating cancer cells [1]. By their innate or acquired ability to resist to current therapies, these cells might be responsible for tumor dissemination and metastasis after therapies [1]. The current hypothesis is that MCCs present features shared with initiating cancer cells but at the moment no specific biomarkers to phenotypically distinguish them are available.

A typical feature of MCCs is the ability to show a metastable phenotype encompassing epithelial and mesenchymal traits [14]. The cell dedifferentiation occurring during epithelial-to-mesenchymal transition (EMT) consists in the transcriptional repression of E-cadherin paralleled by the transcriptional induction of mesenchymal markers, including N-cadherin, vimentin, alpha-smooth muscle actin and fibroblast-specific protein 1 [1]. The transcriptional changes are mediated by a set of transcription factors, Snail, Slug, Twist1 and Zeb, activated by tumor-growth factor β (TGFβ), Notch and Wingless-INT (Wnt) signaling pathways [1]. After seeding cancer cells undergo mesenchymal to epithelial transition (MET), halting migration and allowing proliferation [14]. Since MET is necessary for the metastatic process, it is conceivable that MCC represents a cancer cell in transitional EMT/MET state.

Oxidative stress plays a contributive role in this context, being a driver of malignant transformation and fostering a highly metastatic phenotype [15]. Nevertheless, the exact effect of ROS on metastatic cascade is still controversial. Although, moderate ROS levels sustain tumor development by activation of cell survival, proliferation and migration pathways, the balance of cellular ROS levels is critic, because a high ROS concentration leads to apoptosis of cancer cells. For this reason cancer cells depend on elevated antioxidant capacity, which allows ROS levels without exceed the threshold compromising cell viability [15]. In this light, clinical trials using antioxidants failed, since cancer patients experienced worse prognosis. On the other hand, there are also some studies demonstrating that the inhibition of mitochondrial oxidative stress is able to counteract metastatic cascade.

The higher content of ROS is ensured by increased basal metabolic activity, mitochondrial dysfunction paralleled to hypoxia or mitophagy, uncontrolled cytokines signaling, and oncogene activity. Several known ROS sources, including NOXs, cyclooxygenases, or lipoxygenases, show an intense enzymatic activity contributing to keep ROS levels higher than in normal cells. Different signaling pathways associated with oxidative stress influence metastatic phenotype.

TGF-β increases ROS production by activating the transcription of NOX-2 and -4 and suppresses antioxidant enzymes, leading to a redox imbalance [16]. ROS, in turn, induce/activate TGF-β pathway at different levels and mediate some of its effects. In particular ROS can facilitate the release of the active form of TGF-β from the small and large latent complexes, which immobilize this cytokine in the extracellular matrix [17].

Wnt is a complex signaling pathway that controls cell differentiation and proliferation in embryo and adults by a canonical or non-canonical pathway. Canonical pathway is characterized by the nuclear translocation of β-catenin, while the non-canonical pathway signals through Frizzled receptors and triggers calcium-mediated biological functions including cell polarity and stemness [18]. ROS have an activating impact on Wnt system. Trx-like protein nucleoredoxin negatively regulates the inhibitory activity on the destruction complex deputed to degrade β-catenin. The oxidation of this reductase or its deletion stabilizes β-catenin, thus favoring its transcriptional activity. Wnt pathway itself activates ROS generation and amplifies its signaling activity by a Src-dependent phosphorylation of NOX-1 and subsequent production of H_2_O_2_ [19]. Furthermore alteration of redox state antagonize the effect of Dickkopf 1, an endogenous Wnt inhibitor [18], and stabilize the Frizzled co-receptor Low-density lipoprotein receptor-related protein 5 [20].

Accumulating evidences have suggested that ROS also function as second messengers modulating stem cell self-renewal and differentiation by regulation of intricate signaling networks.

Differently from cancer cells, cancer stem cells produce low amount of ROS [21].

To maintain low level of ROS, the metabolic set point of cancer stem cells is set-up to favor the reductive metabolism and the production of antioxidants. This peculiarity is mainly governed by hypoxia inducible factor (HIF) [22] and NRF-2 systems [22, 23].

HIF induces glycolytic genes and pyruvate dehydrogenase kinase 1, which phosphorylates and inhibits pyruvate dehydrogenase to switch cells from oxidative to glycolytic metabolism. Furthermore it triggers mitophagy [24] and represses oxidative metabolism by inhibiting the fatty acid oxidation and the complex I of electron transport chain [25]. Finally, HIF increased the production of NADPH by activating the mitochondrial catabolism of serine [26] thus increasing the production of antioxidant species.

Interestingly, NRF-2, the master gene of redox system, is often mutated in cancers [27] and is highly expressed in some cancer stem cell types [23]. Similarly to HIF, NRF-2 activates the transcription of glycolytic genes and pyruvate kinase dehydrogenase, thus reducing mitochondrial oxidation and ROS production [28]. Furthermore, NRF-2 might participate indirectly to hepatic oncogenesis as inferred by the impact of loss of PKCλ/ι in hepatocarcinoma. In fact the deletion of this kinase in hepatocytes promotes the onset of hepatocarcinoma linked to increased ROS and NRF2 activation [16].

**Fig. 3:**
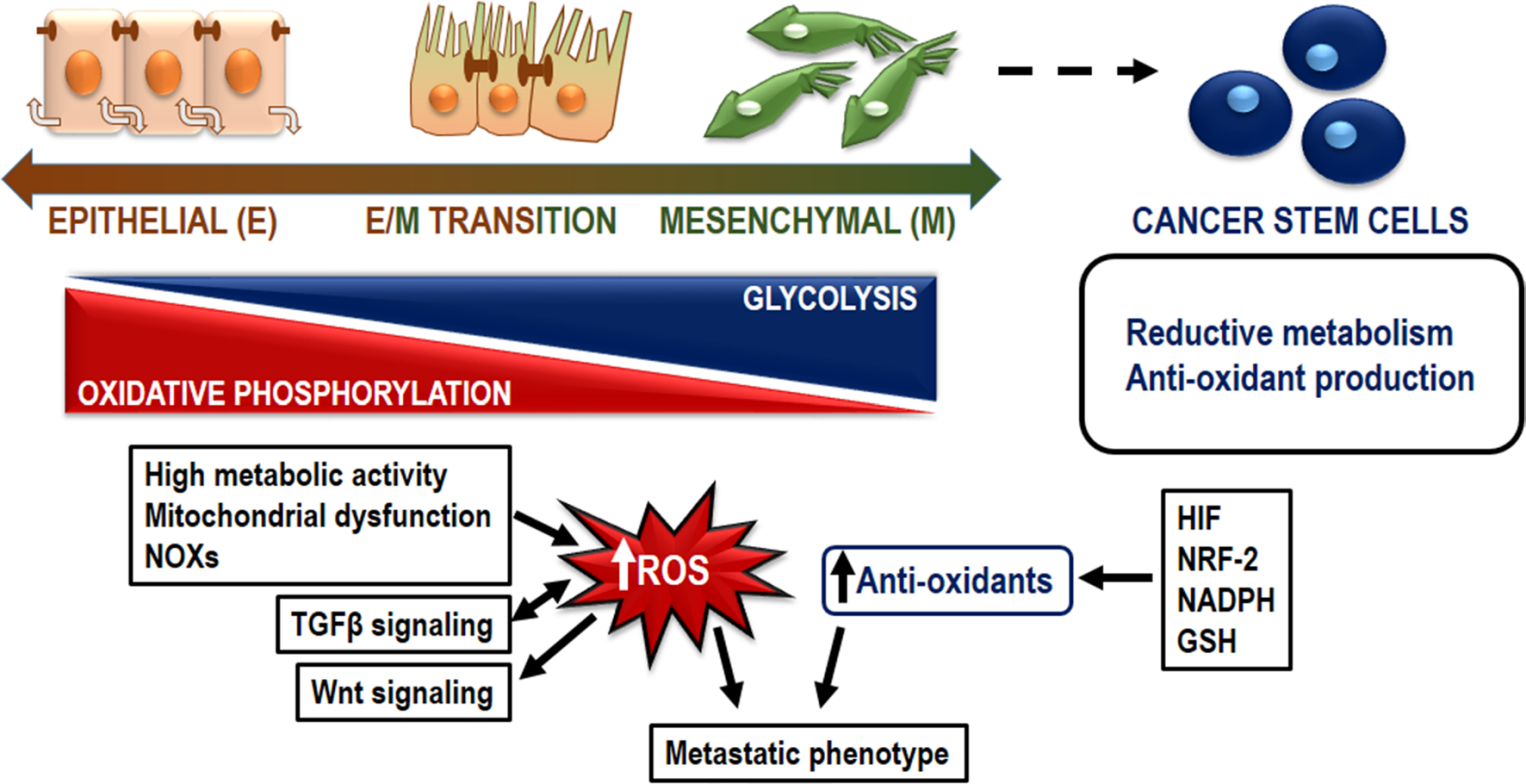
Intrinsic features of dedifferentiation mechanisms adopted by cancer to generate a pool of stem cells sustaining tumor growth. During EMT, epithelial cells de-differentiate into mesenchymal cells. This process is possible by gradual alteration of specific markers and progressive change in metabolic pathways. Epithelial cells obtain energy from oxidative phosphorylation, whereas mesenchymal cells are glycolytic. Mesenchymal cells can then originate cancer stem cells by a further step of dedifferentiation, in which a reductive metabolism is established as well as the production of anti-oxidant species. The high metastatic phenotype is sustained by a fine balance between ROS and antioxidant levels. ROS levels are generated by a high metabolic activity, paralleled by mitochondrial dysfunction, cytokine signaling. TGFβ signaling is both sensitive to and induces ROS. ROS content in MCC induces Wnt signaling. Among active antioxidant systems, HIF, NRF-2, NADPH and GSH play a pivotal role

A holistic and fruitful interpretation of the experimental data available can be obtained by considering the involvement of redox metabolism in metastasis diffusion as a dynamic process finalized to maintain a balanced redox state a little bit shifted towards reducing equivalents finalized to maintain ROS levels consistent with the cellular fitness. The analysis of melanoma patient-derived xenografts elegantly demonstrates that metastatic activity is related to the concomitant increase of ROS production and antioxidant molecules, such as NADPH and GSH [15]. The investigation of redox state related to primary tumors, circulating tumor cells and metastatic sites allowed to demonstrate that MCCs are able to rewire their metabolic profile to withstand the oxidative stress they experience along the metastatic travel. Actually, ROS levels are higher in circulating cells and in metastatic nodules than primary tumors. However, the ratio GSH/oxidized GSH (GSSG) and the amount of NADPH originated from folate pathway are higher in metastasis than the primary lesions indicating that cancer cells undergo an adaptive change to buffer the oxidative stress (Fig. 3).

## The travel of metastatic cancer cells in the bloodstream

To successfully and safely navigate into the blood circulation, extravasate and colonize a distant site from the primary tumors MCCs need the collaboration of other cell types, including endothelial cells (ECs), PMNs and platelets. These cells have specific metabolic peculiarities, which impact on oxidoreductive system and indeed on MCC circulation.

### ROS generation and function in vascular endothelial cells

#### ROS generation

Redox balance is crucial for endothelial fitness and its homeostasis gets worse during life. Endothelial oxidative stress is involved in many pathological events, including vasoconstriction, vascular remodeling, inflammation, angiogenesis and fibrosis [29]. In this light, ECs need ROS for many of their activities, but concomitantly a precise control is required to avoid the detrimental effects of oxidative stress, which might be further facilitated and amplified by the assumption that ECs could directly sense the high O_2_ levels into the blood.

ECs have different systems to generate ROS. Nox-4 and Nox-2 account for the basal tone of ROS whereas Nox-1 and Nox-5 have been shown to mediate both physiological and pathological events in the vascular system [30]. Another mechanism for ROS production relies on the cooperation between NOX and NOS3. Uncoupled NOS3 generates H_2_O_2_ instead of NO when the availability of arginine or the cofactor tetrahydrobipterin is low. Superoxide reacts with NO forming peroxynitrite that further oxidizes tetrahydrobipterin creating a vicious circle and more NOS3 uncoupling [31]. Xanthine oxidoreductase, which catalyzes the reduction of hypoxanthine to uric acid and generates H_2_O_2_ and O_2_, together with the mitochondrial electron transport chain are involved in ROS production during pathological conditions [30]. ROS also regulate the activity of H_2_S, a gas involved in endothelial functions, including angiogenesis, and primarily produced by the trans-sulfuration enzyme cystathionine-γ-lyase. H_2_S exists as hydrosulphide anions (HS^–^) and protons (H^+^) with the remaining third being in the form of H_2_S undissociated gas. The mechanism supporting H_2_S functions requires ROS-dependent activation of polysulphides (reviewed in [32]), which can oxidize cytesine residues in target proteins leading to the formation of disulfide bonds. Furthermore, polysulphides are less polar and more lipophilic than H_2_S facilitating their diffusion in adjacent cells to propagate the activating signal.

Considering the almost unlimited availability of O_2_ and the requirement of a judicious ROS level, ECs have developed different strategies to avoid oxidative stress and in particular they utilize diversified reducing systems. The use of glycolysis to produce ATP instead of mitochondrial respiration is the first method exploited by quiescent and angiogenic ECs to keep ROS production in a narrow and low range [33]. In nascent vessel, glycolysis rate is increased both in migratory tip cell and proliferative stalk cell as compared to quiescent ECs far from the sprouting area [34]. This process is largely correlated with the increased expression of phosphofructokinase-2/fructose-2,6-bisphosphatase-3, which catalyzes the synthesis of fructose-2,6-biphosphate, the allosteric activator of phosphofructokinase-1 [34]. Of note, a recent paper contradicts this vision indicating that both glycolysis and mitochondrial respiration occur during proliferation of non-tip cells [35].

To reduce the risk of oxidative stress, quiescent ECs show a high rate of fatty acid oxidation to fuel tricarboxylic acid cycle (TCA). The activation of this pathway does not support ATP production or biomass synthesis but assures redox homeostasis. The TCA metabolite malate can be exported to the cytosol via the dicarboxylate carrier and oxidized to pyruvate by the NADP-dependent malate dehydrogenase. NADPH in turn supports GSH synthesis resulting in ROS quenching [36]. Of note, the inhibition of carnitine acyltransferase I, the limiting step of mitochondrial fatty acid oxidation fueling TCA, leads to endothelial alteration [36].

**Fig. 4:**
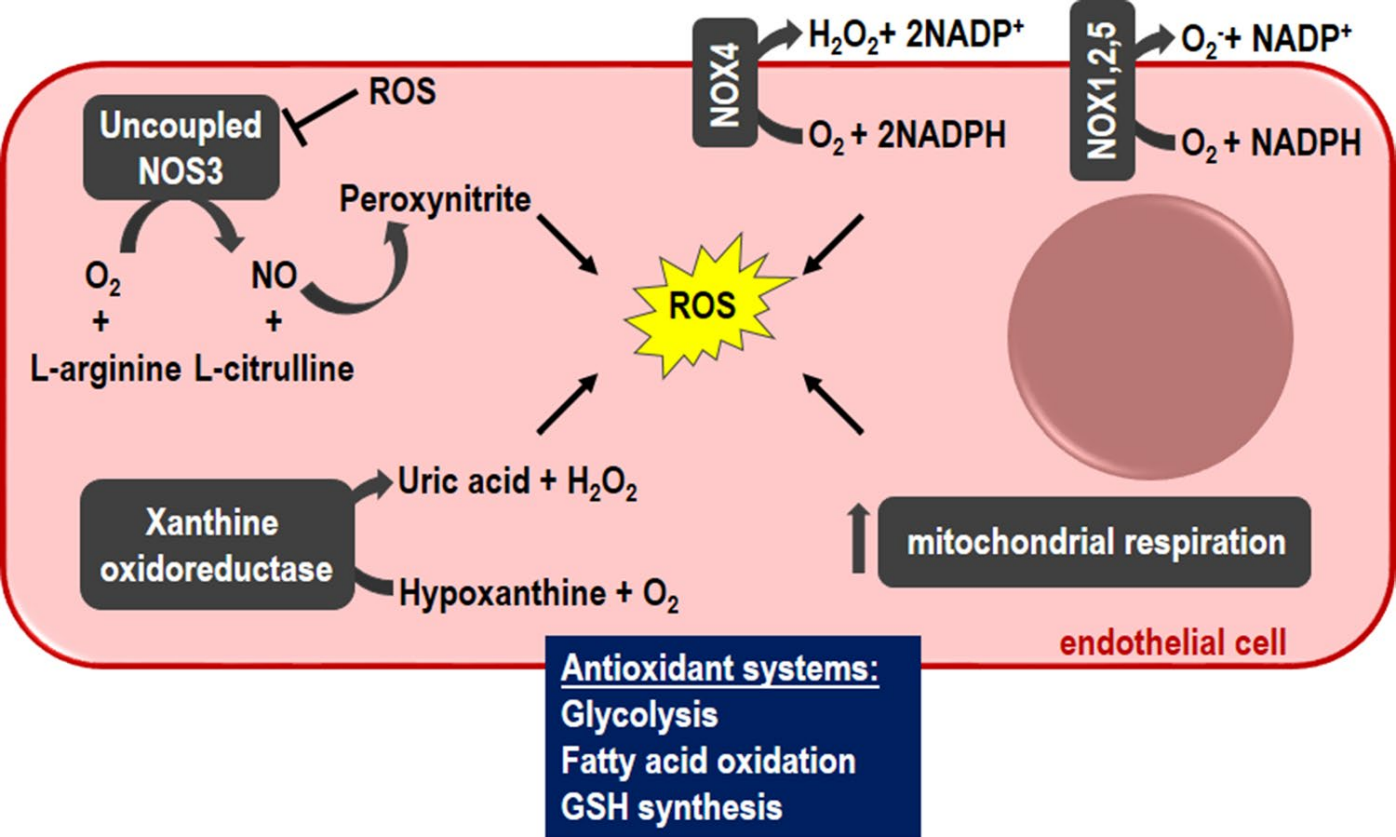
Endothelial ROS sources. ECs generate ROS through several enzymatic systems: NADPH oxidases (NOXs), nitric oxide synthase 3 (NOS3), xanthine oxidoreductase and during mitochondrial respiration. Considering the high EC exposure to O_2_, endothelium developed different reducing systems, including glycolysis, fatty acid oxidation and glutathione (GSH) synthesis

Furthermore, other metabolic pathways contribute to generate an efficient and antioxidant system in ECs. Supplemental Table 1 summarizes the most important effects induced by the alteration of the endothelial reductive pathways to frame the role of vasculature in the metastatic process (Fig. 4).

#### ROS function

**Fig. 5:**
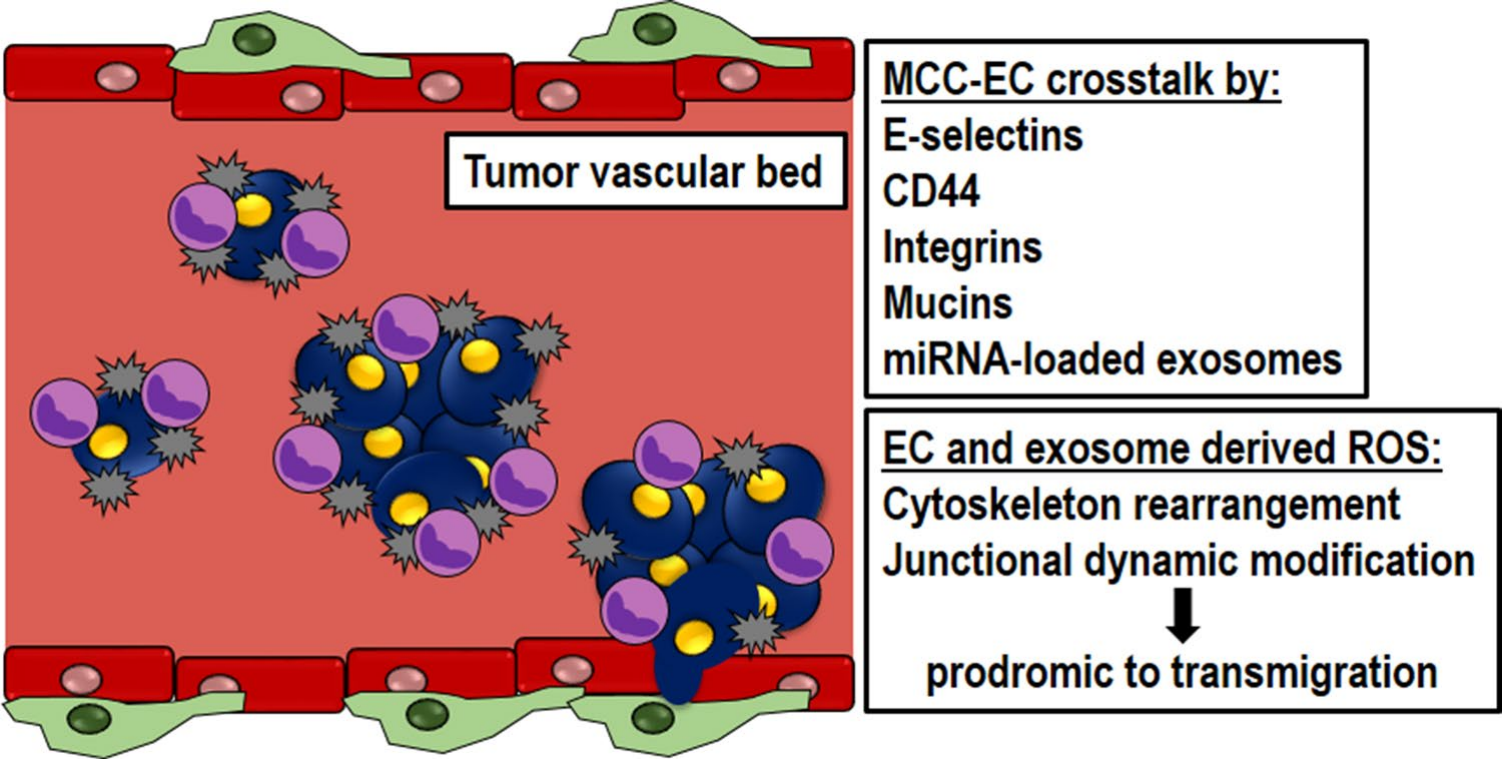
Endothelial role during MCC journey. Endothelium works as a physical barrier to be crossed by MCC and as a support to the formation of the metastatic niche through different signaling pathways sensitive to ROS

In the metastatic process, ECs represent both a physical barrier to be crossed by MCC and a support to the formation of the metastatic niche [6]. Leukocyte extravasation is an operative guideline to examine EC functions engaged by MCC [37] (Fig. 5).

Leukocyte diapedesis occurs at site of tissue injury, where inflammatory cytokines induce the expression of endothelial selectins, which orchestrate the ‘first contact’. Locally accumulated chemokines instruct leukocytes to polarize and acquire a motile phenotype. This step needs the interaction between leukocyte and their endothelial ligands ICAM-1 and VCAM-1. Subsequently, the cells form protrusions to cross endothelial barrier, through EC intercellular junctions or by penetrating EC body [38].

The arrest of MCC on vascular surface depends on the physical restriction of capillaries [37], or on active adhesive processes involving selectins, integrins, cadherins, CD44 and immunoglobulin superfamily receptors [39, 40].

As demonstrated for leukocytes, MCC starts to crawl on endothelial surface by the formation of weak adhesions by the engagement of endothelial E-selectin [41] or CD44 and αvβ3 integrin, as recently reported in vivo [39]. Different modes to stabilize this arrest with higher adhesion strength have been described and exploit integrins, CD44 and mucins [39, 40]. A striking difference between the two processes relies on their specific triggering stimuli: while inflammation represents the onset of leukocyte extravasation, the mechanisms allowing MCC to colonize a specific organ are poorly understood [1, 7, 8]. An emerging concept focuses on the ability of primary tumors to instruct vascular barrier to host MCC by releasing exosomes and specific microRNAs that modify EC permeability [4]. Interestingly, exosomes can directly scavenge or produce ROS but they can also act on ROS indirectly, modifying the ROS content of their target cells [11]. Nevertheless, the role of exosome ROS-related cargo in metastatic cascade is completely unknown.

ROS are produced during leukocyte diapedesis by ECs [42] and regulate their molecular arrangement, which characterizes cytoskeleton and junctional dynamics occurring during leukocytes transmigration (Supplemental Table 2).

### ROS generation and function in polymorphonuclear cells

PMNs, cells of the innate immune system, are recruited at site of infections to phagocytize and kill pathogens. They are professional ROS producers to exert their antimicrobial defense function. PMN phagocytosis or chemo-attraction depends on ROS release, mostly produced by NOX-2 function and by myeloperoxidases, specific enzymes of PMNs. NOX-2 activation and its cooperation with myeloperoxidase occur both outside PMN plasma membrane and in phagosome. The former characterizes PMN activation by soluble inflammatory stimuli (immune-complexes, anaphylatoxins, chemokines and cytokines) with the concomitant release of azurophil granules, containing myeloperoxidase. The latter is typical of phagocytosis, in which PMN membrane embeds the pathogen and fuses with azurophil granules enabling ROS synthesis inside the phagosome [43]. To remove bacteria, PMN release neutrophil extracellular traps (NET), a mesh of nucleic acid, enzymes and antimicrobial agents, whose assembly depends on ROS availability [44]. Specifically, ROS damage the membrane of azurophils granules and nucleus, allowing elastase and myeloperoxidase to enter into nucleus promoting chromatin condensation [45].

### ROS generation and function in platelets

Platelets show a basal production of ROS, which rapidly increases when platelets start to adhere to injured vessel walls, to one another or to other circulating cells [46–48]. This adhesive process is largely mediated by platelet integrins [46], which might undergo post-translational modifications of critical cystein thiol groups. ROS generation is mainly mediated by NOX-1 and NOX-2 and occurs during platelet activation with an enhancing effect. Tumor necrosis factor receptor associated factor-4 binds the cytosolic tail of the collagen receptor glycoprotein VI and recruits at the plasma-membrane the *p47*^*phox*^ subunit of NOX-1 and-2 leading to ROS generation [49].

Besides influencing integrin-mediated adhesive process [46], ROS might modulate the intracellular signaling pathways supporting granule release and activation of polyunsatured fatty acids (e.g. arachidonic acid) cascade [50]. The deletion of Prx2 allows higher levels of ROS and potentiates platelet activation [51]. ROS antagonizes the disaggregating activity mediated by NO, by reducing its activity and forming peroxynitrites [52].

### Polymorphonuclear cells and platelets: fellow travelers of metastatic cancer cells

**Fig. 6:**
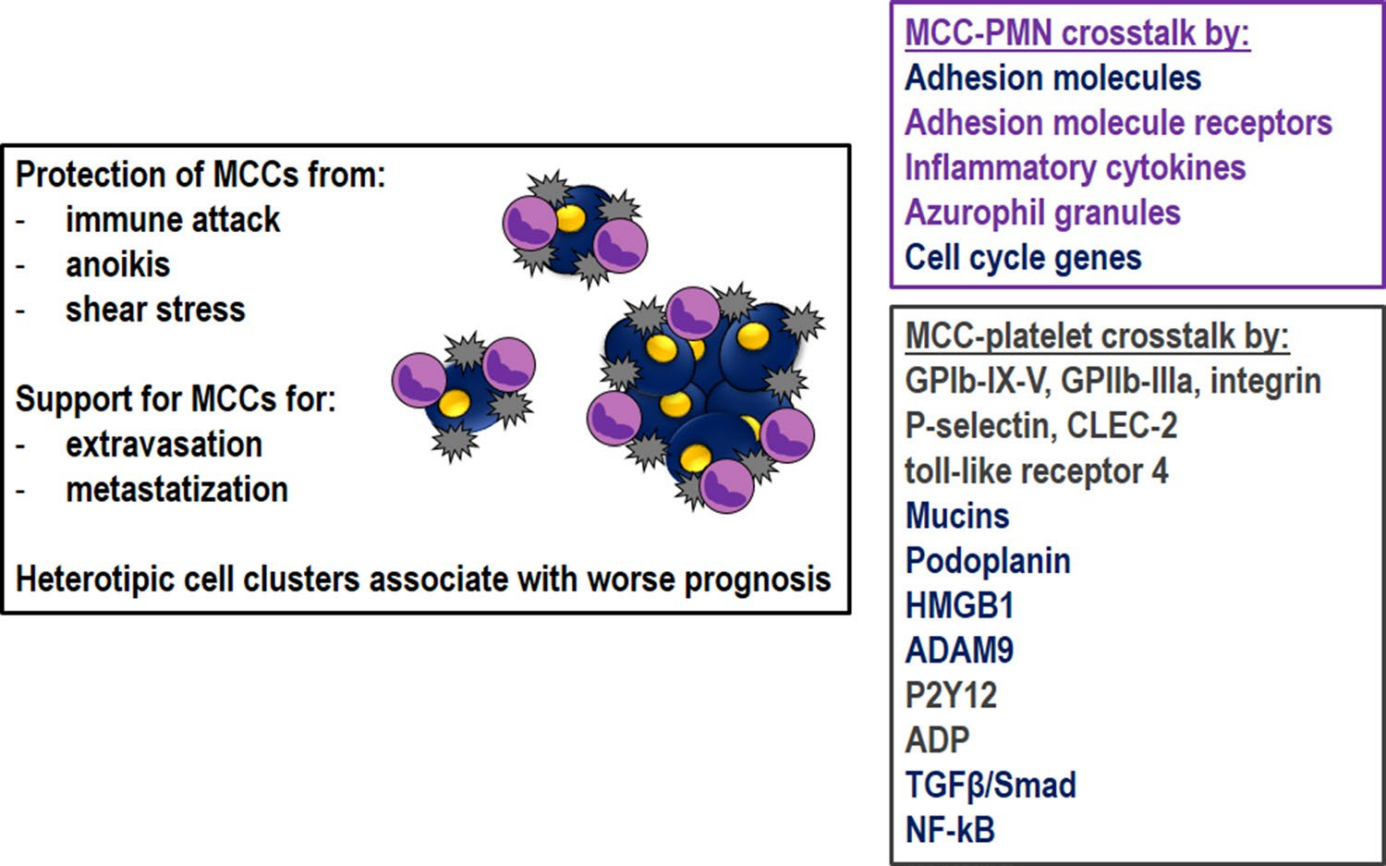
PMN and platelet role during MCC journey. PNMs and platelets protect MCCs from immune cells, anoikis and endothelial shear stress. They support MCCs during extravasation and establishment of the metastatic niche. Different signaling pathways sensitive to ROS are implied in PMN/platelets crosstalk with MCCs

The MCC journey in bloodstream is unsafe. They circulate as single cell or clusters [40]. Indeed, the formation of heterotypic cell clusters with leukocytes or platelets is instrumental to protect MCCs from immune attack, anoikis, and dangerous effects of shear stress. Furthermore, these clusters favor the metastasis extravasation step and their presence has been often linked with a worse prognosis [53]. On the contrary, in vivo depletion of PMNs has anti-metastatic effect [54]. The active redox metabolism of these cells can influence MCC behavior to move in blood circulation and to cross endothelial barrier. Of note, a recent paper highlights the role of ROS in determining the heterotypic PMN-platelets interactions during inflammation-induced microvascular occlusion. The data show that platelet and PMN NOX-2-derived ROS regulate the function of surface receptors GPIbα and αMβ2 integrin, which are instrumental for PMN-platelet adhesion [47]. These results inspire a similar scenario that could occur during the cluster formation of MCCs, leukocytes and platelets (Fig. 6).

#### The role of Polymorphonuclear cells

Increasing evidences indicate that MCCs might circulate in bloodstream surrounded by PMNs [55]. Interestingly, MCCs interacting with PMNs express higher amounts of cell-cycle genes than MCCs traveling alone [55], indicating a strict cooperation between the two cell types. The formation of these clusters is mediated by the expression of adhesive molecules on MCCs (ICAM, VCAM) and their cognate receptors on PMN surface [55]. This *in trans* cell–cell stimulation results in the release of inflammatory cytokines (IL-1β, IL-6) by MCCs that stimulate PMNs [55]. It is plausible that PMNs release their granule contents and in particular azurophil granules, which contain myeloperoxidase able to generate ROS [43]. Of note, ROS regulate cell cycle via phosphorylation and ubiquitination of regulatory molecules, such as the phosphatase Cdc25 and cycle kinase inhibitors [56]. These observations have been recently replicated in vivo by analyzing the effect of ROS on *Xenopus* embryos [57]. In this model mitochondria ROS oscillations parallel cell cycle and recognize as target Cdc25 by changing its phosphorylation levels. The use of ROS synthesis inhibitors results in an impairment in the cycling Cdc25C hyper/hypophosphorylation oscillations and in a deregulation of cell cycle. In general, it is also stimulating to speculate that ROS produced by PMNs, contribute to maintain the stemness metastable phenotype of MCCs. This symbiotic cooperation between MCCs and PMNs is reminiscent to that observed in the parasitic disease leishmaniosis, where parasites are camouflaged within PMNs and use their azurophilic granules to survive [58] (Fig. 6).

Very recently, clusters of MCCs, PMNs and myeloid-derived suppressor cells (MDSC) have been described in melanoma and breast cancer patients [59]. This tripartite alliance results in pro-survival signaling for MCCs. Increased ROS produced by PMN-MDSCs upregulate Notch1 in MMCs through the ROS-NRF-2 axis, thus priming MCCs to respond to Jagged-mediated, PMN-MDSC-driven Notch activation.

#### The role of platelets

Platelets interact with MCCs activating themselves and releasing their granules [60] in blood circulation. Their physical interaction is mainly mediated via GPIb-IX-V, GPIIb-IIIa and tumor cell integrin. However other molecules expressed on platelet surface have been identified and include P-selectin, C-type lectin-like receptor 2 (CLEC-2), α6β1integrin and toll-like receptor 4, which respectively interact with tumor mucins, podoplanin, high-mobility group box 1 and a disintegrin and metalloproteinases 9 (ADAM9) [60]. More recently, it has been described the role of the purinergic receptor P2Y12 on platelet surface and ADP at interface between cancer cells and platelets, which promote ovarian cancer progression [61]. Finally, platelets protect MCCs from high shear forces and from immune cells by molecular processes mainly active on natural killer cells [62].

TGFβ accumulated in platelet α granules and the direct platelet-tumor cell contacts synergistically activate Smad and NF-kB pathways in MCCs, resulting in their transition to an invasive mesenchymal-like phenotype and enhanced metastasis in vivo [63]. A similar effect has been recently reported for ATP and purinergic receptors [64] (Fig. 6). Because ROS might regulate MCC phenotype and platelet activation is associated with ROS production [46–48], it is reasonable to speculate that also ROS-derived platelets participate to the survival of tumor cells, as reported for PMNs [55].

## Metastatic cancer cell seeding at metastatic site

### Metastatic cancer cell extravasation as single cell or homotypic cell clusters

MCC extravasation mirrors leukocyte diapedesis and requires strict and private interactions with ECs. They might extravasate as single cell or as clusters formed at the primary site, during the intravasation step, the travel in the bloodstream or at the secondary site arrest (reviewed in [65]). The MCC extravasation occurs by paracellular migration through the EC junctions [66] or by the less explored trans-cellular route from the apical to the basolateral side of ECs [65]. How MCCs select the way to migrate is poorly known and seems to depend on the cancer cell types [40]. In both modalities, ECs show extending filopodia-like processes, which might guide invading cells towards low resistance points. As well described for leukocyte diapedesis, the paracellular migration of MCCs requires the dismantling of *adherens* junctions and a retraction of ECs [66]. It is plausible to envisage that soluble molecules released by MCCs or by companion cells trigger a small GTPase-mediated cascade leading to the phosphorylation of myosin light chain and the subsequent actomyosin-mediated tension of ECs. MCCs might also activate Src/proline rich tyrosine kinase 2 pathway, which induces phosphorylation and disassembly of the VE-cadherin/β-catenin complex and therefore induces EC junction opening [66]. All these intracellular mechanisms are largely influenced by the presence of ROS. Interestingly, MCCs adapt their cytoskeleton to cross EC barrier and form invadopodia, which are dynamic actin-rich protrusive structures regulated by Rac and Rho small GTPases, capable of degrading the extracellular matrix and to guide extravasation [67]. Invadopodia formation relies on the presence of a family of adaptor named Tks, which has a significant homology for *p47*^*phox*^ subunit of NOX [68]. Importantly, the total levels of ROS generated by cancer cells decreases considerably after silencing of Tks [68], indicating that endogenous Tks proteins might be required for ROS generation in cancer cells and most probably in invadopodia, where they contribute to actin dynamics.

Alternatively, extravasation occurs after MCC arresting on EC surface and their intravascular proliferation without penetrating the capillary wall. The tumor mass then mechanically disrupts capillaries leading to MCC invasion [69]. Finally, some tumor cells might induce EC necroptosis followed by MCC extravasation and metastasis. MCCs activate this programmed necrosis by triggering a signaling pathway involving TGF-β-activated kinase 1 or receptor-interacting serine/threonine-protein kinase 1 [70]. The role of ROS in regulating the signaling pathways sustaining necroptosis is largely unknown and the few data available are contradicting. Actually, it was observed both an increased and a decreased ROS production respectively in TGF-β-activated kinase 1-deficient PMNs [71] and epithelial cells [72].

### Metastatic cancer cell extravasation as heterotypic cell clusters

PMNs and platelets surrounding MCCs are not only limited to serve as bodyguard during their travel in the circulation, but they are instrumental to cross endothelial wall. PMNs adherent on endothelial layer stop MCCs on the site of possible colonization by favoring their seeding [73]. PMN ROS are involved in the production of NET, which surrounds MCCs, thereby favors adhesion and promotes metastasis. The role of ROS in NET production is not necessarily connected with an inflammatory status. In cancer patients the albumin reservoir of free thiol is reduced with a reduced ROS scavenger activity. Therefore, ROS might accumulate within PMNs leading to NETosis and seeding of hematogenous metastases [74].

In vivo experimental models have demonstrated that MCC adhesion to ECs is increased by platelets [75]. Furthermore, ADP or ATP released by cancer-activated platelets stimulated P2Y purinergic receptors to promote an increased vascular permeability, most probably by acting on VE-cadherin/β–catenin system, and tumor cell transmigration [76]. A further mechanism exploited by platelet-MCC clusters at endothelial surface relies on leukocyte recruitment. Activated platelets, being in contact with MCCs, release CXCL5 and CXCL7 chemokines and recruit leucocytes, which in turn contribute to MCC extravasation [77].

### The end of the journey: the metastatic niche

**Fig. 7:**
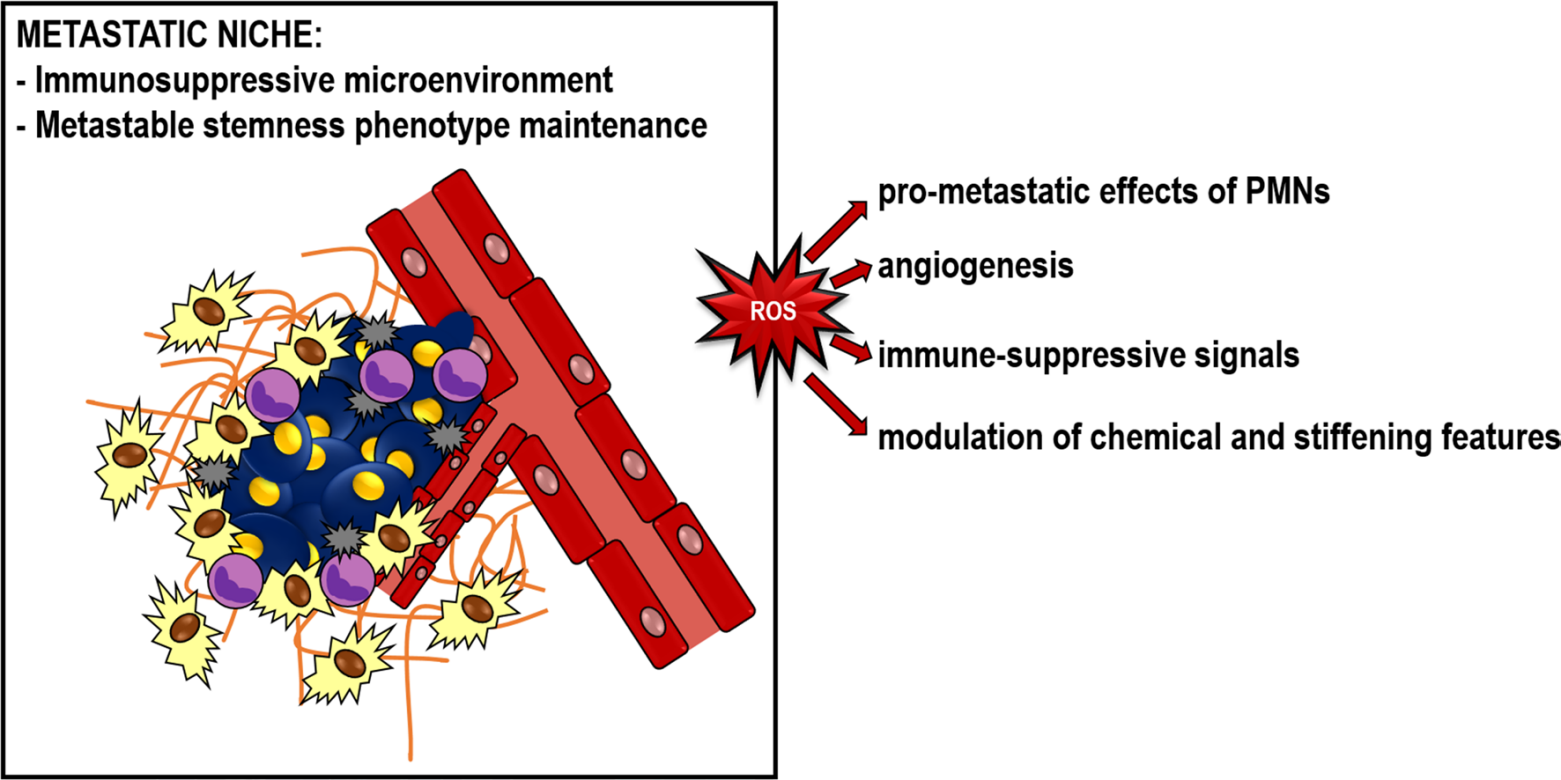
Establishment of the metastatic niche. The metastatic niche is characterized by the presence of different signaling pathways sustaining an immunosuppressive microenvironment and a metastable stemness phenotype, which allow dormancy and activation of metastatic cells. ROS influence metastatic niche features

After crossing the vascular wall, MCCs organize themselves to colonize the new organ starting from the “metastatic niches”, which are reminiscent of the stem cell niche firstly described in hematopoiesis. Features of the metastatic niche show a dynamic behavior alongside disease progression as well as the mechanism sustaining its formation, that precede MCC seeding under the influence of molecules released by the primary tumor (see above) or molecules becoming active on cancer cell arrival [6]. Metastatic niche is formed by ECs, infiltrating leukocytes and fibroblasts and in some tissues by specialized cells including astrocytes in brain, Kupffer cells in liver and osteoblasts in bone. The niche exerts many functions to support disease progression including the protection of tumor cells from the attack of immune system by creating an immunosuppressive microenvironment, and the maintenance of the metastable stemness phenotype in dormant state. In these conditions, cancer cells might remain in a quiescent state for years and decades and form the micro metastases. Unpredictable signals can trigger the breakout of latency, characterized by the proliferative licensing engaged by pro-survival signals and vascular angiogenesis, which awaken MCCs from the dormant state. These activities are mediated by a wide array of soluble molecules, including growth factors, cytokines and prostanoids, the physic-chemical features of extracellular matrix, and the contact mediated intercellular communications. As final result of this complex interplay, cancer cells start an overt growth and an invasion of the tissue near the niche becoming manifest and clinically relevant metastasis [6]. ROS produced inside the metastatic niche from different cellular sources negatively or positively regulate the metastatic cascade (Fig. 7).

#### ROS and polymorphonuclear cells

PMNs represent a relevant source of ROS. In a preclinical model of lung carcinoma, cancer cells signal to bone-resident osteoblasts through the systemic release of the soluble receptor for advanced glycation end. Stimulated osteoblasts in turn promote the generation of a specific subset of tumorigenic PMNs (SiglecF^hi^) with cancer promoting functions mediated by macrophage M2 polarization and ROS mediated immune suppression [78]. Interestingly at the metastatic niche ROS might increase the pro-metastatic effect of PMNs by increasing the production of ferritin [79], which is a powerful mitogen for cancer cells. The presence of PMNs in the niche and their ability to extrude NET is essential for the omental metastasis of ovarian cancer. In fact the deletion of the peptidylarginine deaminase 4, an enzyme that cooperates with NOX to determine chromatin decondesation during NET formation, blocks omental metastasis [80]. NET production is also required for awakening dormant cancer through NET-associated proteases, which remodel laminin thus promoting the proliferation of dormant cancer cells [81]. On the contrary, ROS released by PMNs can exert an anti-tumor activity by suppressing the pro-tumoral IL-17^+^ γδ T cells [82].

#### ROS and angiogenesis

The first demonstration of the essential role of ECs in maintaining stemness features of tumor cells has been provided in brain tumors, where anti-angiogenic therapies altered the perivascular niche hosting cancer cells and reduced the cancer stem cell population [83]. It is definitely established that vascular supply is crucial for metastasis embedding and it occurs by generation of new vessels [8] or by vessel co-option, a process whereby tumor cells proliferate around capillaries.

The role of ROS in angiogenic process has been inferred by the use of anti-oxidant molecules that inhibit angiogenesis. This notion is further supported by mouse genetic studies, showing that the deletion of genes involved in redox metabolism, such as catalase or NOX, has a deep impact on sprouting angiogenesis and vascular remodeling (reviewed in [84]). ROS influence angiogenesis by acting on Vascular-Endothelial growth factor (VEGF) pathway. Specifically, ROS might induce VEGF production by different cell types, such as ECs, vascular smooth muscle cells, fibroblasts, pericytes and macrophages, and VEGF itself activates ROS production by involving both cytosolic and mitochondrial production [85]. Furthermore, ROS increase the expression of VEGF receptor 2 on ECs, and promote its activation and downstream signals [85]. Besides VEGF pathway, ROS might exploit other systems to promote angiogenesis. For instance, end products of lipid oxidation are recognized by Toll-like receptor 2 on ECs leading to angiogenic response [86].

#### ROS and immune suppressive function

Metastatic niche is characterized by immune suppressive conditions that induce MCCs to escape from immune surveillance. This immunotolerance state is promoted by MDSCs, regulatory T cells (T_reg_), and M2-polarized macrophages [87]. For instance, tumor cells induce the expression of tumor necrosis factor-α-induced protein 8-like 2 in myeloid cells through ROS. This protein in turn increases the expression of pro-tumoral mediators while inhibiting the expression of antitumoral mediators. ROS prevent also the differentiation of MDSC into mature myeloid cells, thus maintaining their immune-suppressive activities [88]. ROS themselves represent a MDSC weapon to suppress T cells, by specific effects on CD8 and CD3 molecules and T cell receptor [89]. ROS have a similar function on T_reg_. Actually, it has been reported that macrophages lacking NOX displayed hampered T_reg_ induction and T-cell suppression [90]. Interestingly, X-linked chronic granulomatous disease gp91^*phox*^- deficient patients, which have an impairment in ROS production, showed a reduced number of circulating T_reg_ [91]. As reported for MDSC, T_reg_ exert their suppressive activity by releasing exosomes containing NOX-2. These microvesicles are up-taken by T-cells where they produce ROS, which inhibit T cell receptor signal transduction by reducing phosphorylation of the T cell receptor-associated tyrosine kinase ZAP70 [92].

Depending on the content of intracellular GSH, the M1 and M2 macrophages, which respectively have an anti- and pro-tumoral function, are characterized as oxidative and reductive macrophages [93]. In contrast to M1 macrophages, M2 polarization is connected with a reduced ROS activity [93]. However, these results conflict with the observation that blocking O^2−^ synthesis impaired M2 polarization [94].

#### ROS and extracellular matrix

The chemical and stiffening features of metastatic niche are constantly remodeled to follow the needs of MCCs. An example is the release from primary tumor of lysyl oxidase, which in the lung cross-links collagen IV to increase the anchorage of breast MCCs [95]. Niche stromal cells release metalloproteases required to remodel extracellular matrix and stiffness changes regulating the dormant state of MCCs [96]. Finally, extracellular matrix stiffening mechano-activates glycolysis and glutamine metabolism within the tumor niche. A symbiotic metabolic collaboration relies on the exchange between aspartate and glutamate respectively from fibroblasts and cancer cells, which sustain tumor growth and balance redox state in fibroblasts to promote the extracellular matrix remodeling [97].

The chemical nature of molecular constituents of extracellular matrix, including proteins (collagens, fibronectin, laminins) and proteoglycans, is characterized by regions suitable for redox-regulation by ROS [98]. For instance, the mature structure of collagen and fibronectin requires posttranslational oxidative modifications that might be catalyzed by specific oxidases or by ROS generated in the microenvironment [98]. Metalloprotease activity is enhanced by oxidative activation of an internal inhibitory thiol group and the promoter of metalloprotease-1 is sensitive to redox balance. Redox-dependent expression has been shown to be regulated through Ets-1 and AP-1 DNA consensus binding site of the promoter in response to oxidant-dependent activity of MAP kinase [99]. An epigenetic ROS-dependent mechanism further amplifies metalloprotease-1 expression. High levels of ROS promote HDAC2 degradation, thereby keeping the metalloprotease-1 gene promoter accessible to transcription factors [100].

## Concluding remarks

Hereby, we reviewed features and mechanisms harnessing cell detachment from the primary tumor able to circulate into the bloodstream and ultimately colonize a distant organ establishing a metastatic niche, which could remain quiescent for years and become manifest and clinically relevant all at once by unpredictable signals. All the aspects related to the metastatic program have been discussed focusing on how ROS can influence specific features of MCCs, which favor migratory and stem phenotype acquisition allowing MCC circulation into the blood and seeding at the metastatic site of distant organs, where metastatic niche develops. Specifically, ROS might influence the development of MCCs within the primary tumors with intermediate features between epithelial and mesenchymal phenotype and gain of stemness and migratory skills favoring circulation into the vasculature. The journey of MCCs into the blood is closely followed by ECs, PMNs, and platelets, whose functions depends on ROS availability. Once reached the site of metastasis, either homotypic or heterotypic clusters might give rise to a metastatic niche with specific structural and metabolic characteristics, able to sustain metastasis survival and protect from immune system surveillance.

## Supplementary Information

Below is the link to the electronic supplementary material.

Supplementary file1 (PDF 99 kb)

Supplementary file2 (DOCX 89 kb)
