# Correction to: The role of redox system in metastasis formation

**DOI:** 10.1007/s10456-021-09806-5

**Published:** 2021-07-08

**Authors:** Chiara Cencioni, Valentina Comunanza, Emanuele Middonti, Edoardo Vallariello, Federico Bussolino

**Affiliations:** 1 Institute for Systems Analysis and Computer Science “A. Ruberti”, National Research Council (IASI-CNR), 00185 Rome, Italy; 2 Department of Oncology, University of Torino, 10043 Orbassano, Italy; 3 Candiolo Cancer Institute, FPO - IRCCS, 10060 Candiolo, Italy

## Correction to: Angiogenesis 10.1007/s10456-021-09779-5

In the original publication, the affiliation information of the authors was processed incorrectly. It has been updated in this correction.

