# Supplementary material for: The role of redox system in metastasis formation
Source: Angiogenesis. 2021 Apr 28;24(3):435–50. doi: 10.1007/s10456-021-09779-5 (PMC8292271; doi:10.1007/s10456-021-09779-5)
Supplement: Supplementary file 1 — Supplementary file1 (PDF 99 kb) [file 10456_2021_9779_MOESM1_ESM.pdf]

IS THERE A ROLE OF REDOX STATE IN THE EXTRAVASATION STEP OF METASTASTATIC CASCADE?

Chiara Cencioni[1*], Valentina Comunanza[2,3*], Emanuele Middonti[2,3], Edoardo Vallariello[2,3], Federico Bussolino[2,3]

[1] National Research Council (CNR). Institute for Systems Analysis and Computer Science, 00185 Rome, Italy

[2] Department of Oncology. University of Torino. 10043 Orbassano. Italy

[3] Candiolo Cancer Institute – IRCCS-FPO. 10063 Candiolo. Italy

Correspondence to: Federico Bussolino. MD, PhD

Strada Provinciale di Piobesi 142, Km 3.95

10060 Candiolo (Italy)

Phone: +390119933347

Fax: +39099933524

Key Words: neutrophils, endothelial cells, metastatic cancer cells, platelets

*Equally contributed

Supplemental Table 1. Exemplificative biological effects involved in metastatic process rely on the manipulation of antioxidant systems in ECs

Reducing pathway	Topic	Possible role in metastatic process	Ref.
**Thioredoxins**			
Trx-interacting protein	This protein is required for VEGFR2 catalytic activity via the inhibitory S-glutationylation of low molecular weigh tyrosine phosphatase associated with VEGFR2	Metastatic niche	[1]
Trx-interacting protein	The association between this protein with NLRP3 inflammasome induced by ROS promote endothelial inflammatory response	MCC extravasation	[2]
Trx-interacting protein	This protein is involved in endothelial – leukocyte adhesion	MCC extravasation	[3,4]
Trx reductase	The mitochondrial isoform is required to maintain anti-inflammatory properties of ECs and their angiogenic potential	MCC extravasation; Metastatic niche	[3]
**Peroxideroxins**			
Prx1	It promotes VEGF production in ECs and trigger an autocrine loop		[5]
Prx1	It prevents inflammatory response in early atherosclerosis		[6]
Prx2	It prevents inflammatory response in atherosclerosis		[7]
Prx2	It maintains in reduced state VEGFR2 enabling its activity		[8]
Prx6	It maintains EC barrier function and its deletion increase vasopermeability		[9,10]
**GSH & PPP**			
NADPH	It is required for NOS3 activity and indeed the control of vasopermeability and vascular tone		[11]
Glucose-6-phosphate dehydrogenase (G6PDH)	It is the limiting step of PPP and its genetic manipulation alters angiogenic response of ECs		[12]
G6PDH	GSPDH deficiency favours leukocyte adhesion		[13]
GSH	GSH regulates eNOS production induced by the adhesion molecule ICAM-1		[14,15]
GSH	S-glutathionylation of regulation of the low molecular weight protein tyrosine phosphatase and focal adhesion kinase, which are key mediators of VEGF-mediated cell migration		[16]
GSH	The ratio of GSH:GSSG decrease enhances protein S-glutathionylation, increased ROS, and enhanced VEGFR2 activation		[17]
GSH	The redox-sensitive Ca2+ store maintenance via *S*-glutathione adducts on the key SERCA 2 Cys-674 thiol is required for normal angiogenic EC function		[18]

GSH	By up-regulating Nrf2, GSH maintains EC barrier function and prevents inflammatory response		[19,20]
GSH	S-glutathionylation of NOSIII induces NO uncoupling in ECs		[21]
Glutaredoxin-1	Glutaredoxin-1 is an enzyme that removes GSH from S-glutathionylated proteins. It regulates VEGF pathway.		[22]
GSH peroxidase	The deletion of this enzyme promotes NOS uncoupling and enhances ROS production		[23,24]

Supplemental Table 2. Effects of ROS on the molecular response of ECs to leukocytes extravasation

**Endothelial response to leukocyte extravasation**	**Ref.**	**ROS effects**	**Ref.**
Activation of RhoA-Rho kinase-myosin light chain kinase pathway to regulate actin organization occurring during neutrophil diapedesis	[25-27]	Rho-kinase needs prior activation of G-protein RhoA, which in turn requires activation of guanine nucleotide exchange factors (GEFs). ROS may modulate this pathway by activating the up-stream Src kinase, which activate RhoA, or by a direct effect on RhoA and GEF.	[28-30]
Membrane trafficking at EC border where leukocyte are transmigrating depends on microtubule stabilization and kinesin. Microtubule dynamics is regulated by RhoA and the specific Rho GTPase-activating protein (ARHGAP18).	[31,32]	RhoA is activated by ROS and counteracted by GAP proteins, which accelerate GTP hydrolysis and convert RhoA to the inactive state. Data on the role of ROS on RhoA-GAP proteins are missing. However ROS may activate GAPs linked to other GTPase proteins.	[28,33,34]
Tyr phosphorylation of VE-cadherin destabilizes *adherens junction* and favors leukocyte extravasation.	[35-37]	The tyrosine phosphorylation status of VE-cadherin is regulated by the phosphatases SHP2 and VE-PTP and the kinases Src and proline rich tyrosine kinase 2, which are respectively inhibited and activated by ROS.	[38,39]
Leukocyte extravasation depends on phosphatidylinositol kinase	[40]	ROS levels are associated with an increase in the signaling of phosphoinositide-3,4,5-trisphosphate via oxidation of PTEN and subsequent activation of phosphatidylinositol kinase	[41,42]
During leukocyte diapdesis endothelial CD99 activates protein kinase A and forms a complex with the A-kinase anchoring protein ezrin, and the soluble adenylyl cyclase. Protein kinase A stimulates membrane trafficking from the lateral border recycling compartment to sites of transmigration.	[43]	ROS may activate protein kinase A and change the subcellular localization of ezrin.	[44,45]
Transient receptor potential canonical 6 (TRPC6) calcium channel controls lateral border recycling compartment trafficking and thus control leukocyte extravasation.	[46]	ROS activate TRPC6 and promotes TRPC6 trafficking to the plasma membrane. On the other hands they inhibit TRPC6 expression.	[47,48]
During inflammation Pannexin 1 channels release ATP, which in autocrine manner stimulates purinergic receptors and VCAM1 expression, thus favoring leukocyte extravasation.	[49]	S-nitrosylation impairs Pannexin 1 channel function	[50]

REFERENCES

1. Abdelsaid M, Matragoon S, El-Remessy A (2013) Thioredoxin- interacting protein expression is required for VEGF mediated angiogenic signal in endothelial cells. Antioxid Redox Signal 19:2199-2212

2. Yin Y, Zhou Z, Liu W, Chang Q, Sun G, Dai Y (2017) Vascular endothelial cells senescence is associated with NOD-like receptor family pyrin domain-containing 3 (NLRP3) inflammasome activation via reactive oxygen species (ROS)/thioredoxin-interacting protein (TXNIP) pathway. Int J Biochem Cell Biol 84:22-34. doi:10.1016/j.biocel.2017.01.001

3. Wang XQ, Nigro P, World C, Fujiwara K, Yan C, Berk BC (2012) Thioredoxin interacting protein promotes endothelial cell inflammation in response to disturbed flow by increasing leukocyte adhesion and repressing Kruppel-like factor 2. Circ Res 110 (4):560-568. doi:10.1161/CIRCRESAHA.111.256362

4. World C, Spindel ON, Berk BC (2011) Thioredoxin-interacting protein mediates TRX1 translocation to the plasma membrane in response to tumor necrosis factor-α: a key mechanism for vascular endothelial growth factor receptor-2 transactivation by reactive oxygen species. Arterioscler Thromb Vasc Biol 31 (8):1890-1897. doi:10.1161/ATVBAHA.111.226340

5. Riddell JR, Maier P, Sass SN, Moser MT, Foster BA, Gollnick SO (2012) Peroxiredoxin 1 stimulates endothelial cell expression of VEGF via TLR4 dependent activation of HIF-1α. PLoS One 7 (11):e50394. doi:10.1371/journal.pone.0050394

6. Kisucka J, Chauhan AK, Patten IS, Yesilaltay A, Neumann C, Van Etten RA, Krieger M, Wagner DD (2008) Peroxiredoxin1 prevents excessive endothelial activation and early atherosclerosis. Circ Res 103 (6):598-605. doi:10.1161/CIRCRESAHA.108.174870

7. Park JG, Yoo JY, Jeong SJ, Choi JH, Lee MR, Lee MN, Hwa Lee J, Kim HC, Jo H, Yu DY, Kang SW, Rhee SG, Lee MH, Oh GT (2011) Peroxiredoxin 2 deficiency exacerbates atherosclerosis in apolipoprotein E-deficient mice. Circ Res 109 (7):739-749. doi:10.1161/CIRCRESAHA.111.245530

8. Kang DH, Lee DJ, Lee KW, Park YS, Lee JY, Lee SH, Koh YJ, Koh GY, Choi C, Yu DY, Kim J, Kang SW (2011) Peroxiredoxin II is an essential antioxidant enzyme that prevents the oxidative inactivation of VEGF receptor-2 in vascular endothelial cells. Mol Cell 44 (4):545-558. doi:10.1016/j.molcel.2011.08.040

9. Huang H, Lennikov A, Saddala M, Gozal D, Grab D, Khalyfa A, Fan L (2019) Placental growth factor negatively regulates retinal endothelial cell barrier function through suppression of glucose-6-phosphate dehydrogenase and antioxidant defense systems. Faseb J. doi:10.1096/fj.201901353R

10. Kümin A, Schäfer M, Epp N, Bugnon P, Born-Berclaz C, Oxenius A, Klippel A, Bloch W, Werner S (2007) Peroxiredoxin 6 is required for blood vessel integrity in wounded skin. J Cell Biol 179 (4):747-760. doi:10.1083/jcb.200706090

11. Sies H, Berndt C, Jones DP (2017) Oxidative Stress. Annu Rev Biochem 86:715-748. doi:10.1146/annurev-biochem-061516-0450371

2. Leopold JA, Walker J, Scribner AW, Voetsch B, Zhang YY, Loscalzo AJ, Stanton RC, Loscalzo J (2003) Glucose-6-phosphate dehydrogenase modulates vascular endothelial growth factor-mediated angiogenesis. J Biol Chem 278 (34):32100-32106. doi:10.1074/jbc.M301293200

13. Parsanathan R, Jain SK (2019) Glucose-6-phosphate dehydrogenase deficiency increases cell adhesion molecules and activates human monocyte-endothelial cell adhesion: Protective role of l-cysteine. Arch Biochem Biophys 663:11-21. doi:10.1016/j.abb.2018.12.023

14. Langston W, Chidlow JH, Booth BA, Barlow SC, Lefer DJ, Patel RP, Kevil CG (2007) Regulation of endothelial glutathione by ICAM-1 governs VEGF-A-mediated eNOS activity and angiogenesis. Free Radic Biol Med 42 (5):720-729. doi:10.1016/j.freeradbiomed.2006.12.010

15. Pattillo CB, Pardue S, Shen X, Fang K, Langston W, Jourd'heuil D, Kavanagh TJ, Patel RP, Kevil CG (2010) ICAM-1 cytoplasmic tail regulates endothelial glutathione synthesis through a NOX4/PI3-kinase-dependent pathway. Free Radic Biol Med 49 (6):1119-1128. doi:10.1016/j.freeradbiomed.2010.06.030

16. Abdelsaid MA, El-Remessy AB (2012) S-glutathionylation of LMW-PTP regulates VEGF-mediated FAK activation and endothelial cell migration. J Cell Sci 125 (Pt 20):4751-4760. doi:10.1242/jcs.103481

17. Prasai PK, Shrestha B, Orr AW, Pattillo CB (2018) Decreases in GSH:GSSG activate vascular endothelial growth factor receptor 2 (VEGFR2) in human aortic endothelial cells. Redox Biol 19:22-27. doi:10.1016/j.redox.2018.07.015

18. Thompson MD, Mei Y, Weisbrod RM, Silver M, Shukla PC, Bolotina VM, Cohen RA, Tong X (2014) Glutathione adducts on sarcoplasmic/endoplasmic reticulum Ca2+ ATPase Cys-674 regulate endothelial cell calcium stores and angiogenic function as well as promote ischemic blood flow recovery. J Biol Chem 289 (29):19907-19916. doi:10.1074/jbc.M114.554451

19. Song J, Kang SM, Lee WT, Park KA, Lee KM, Lee JE (2014) Glutathione protects brain endothelial cells from hydrogen peroxide-induced oxidative stress by increasing nrf2 expression. Exp Neurobiol 23 (1):93-103. doi:10.5607/en.2014.23.1.93

20. Speciale A, Anwar S, Ricciardi E, Chirafisi J, Saija A, Cimino F (2011) Cellular adaptive response to glutathione depletion modulates endothelial dysfunction triggered by TNF-α. Toxicol Lett 207 (3):291-297. doi:10.1016/j.toxlet.2011.09.017

21. Chen CA, Wang TY, Varadharaj S, Reyes LA, Hemann C, Talukder MA, Chen YR, Druhan LJ, Zweier JL (2010) S-glutathionylation uncouples eNOS and regulates its cellular and vascular function. Nature 468 (7327):1115-1118. doi:10.1038/nature09599

22. Murdoch CE, Shuler M, Haeussler DJ, Kikuchi R, Bearelly P, Han J, Watanabe Y, Fuster JJ, Walsh K, Ho YS, Bachschmid MM, Cohen RA, Matsui R (2014) Glutaredoxin-1

up-regulation induces soluble vascular endothelial growth factor receptor 1, attenuating post-ischemia limb revascularization. J Biol Chem 289 (12):8633-8644. doi:10.1074/jbc.M113.517219

23. Oelze M, Kröller-Schön S, Steven S, Lubos E, Doppler C, Hausding M, Tobias S, Brochhausen C, Li H, Torzewski M, Wenzel P, Bachschmid M, Lackner KJ, Schulz E, Münzel T, Daiber A (2014) Glutathione peroxidase-1 deficiency potentiates dysregulatory modifications of endothelial nitric oxide synthase and vascular dysfunction in aging. Hypertension 63 (2):390-396. doi:10.1161/HYPERTENSIONAHA.113.01602

24. Chen CA, De Pascali F, Basye A, Hemann C, Zweier JL (2013) Redox modulation of endothelial nitric oxide synthase by glutaredoxin-1 through reversible oxidative post-translational modification. Biochemistry 52 (38):6712-6723. doi:10.1021/bi400404s

25. Stroka KM, Aranda-Espinoza H (2011) Endothelial cell substrate stiffness influences neutrophil transmigration via myosin light chain kinase-dependent cell contraction. Blood 118 (6):1632-1640. doi:10.1182/blood-2010-11-321125

26. Etienne-Manneville S, Manneville JB, Adamson P, Wilbourn B, Greenwood J, Couraud PO (2000) ICAM-1-coupled cytoskeletal rearrangements and transendothelial lymphocyte migration involve intracellular calcium signaling in brain endothelial cell lines. J Immunol 165 (6):3375-3383. doi:10.4049/jimmunol.165.6.3375

27. Wójciak-Stothard B, Williams L, Ridley AJ (1999) Monocyte adhesion and spreading on human endothelial cells is dependent on Rho-regulated receptor clustering. J Cell Biol 145 (6):1293-1307. doi:10.1083/jcb.145.6.1293

28. MacKay CE, Shaifta Y, Snetkov VV, Francois AA, Ward JPT, Knock GA (2017) ROS-dependent activation of RhoA/Rho-kinase in pulmonary artery: Role of Src-family kinases and ARHGEF1. Free Radic Biol Med 110:316-331. doi:10.1016/j.freeradbiomed.2017.06.022

29. Aghajanian A, Wittchen ES, Campbell SL, Burridge K (2009) Direct activation of RhoA by reactive oxygen species requires a redox-sensitive motif. PLoS One 4 (11):e8045. doi:10.1371/journal.pone.0008045

30. Chen Z, Guo L, Hadas J, Gutowski S, Sprang SR, Sternweis PC (2012) Activation of p115-RhoGEF requires direct association of Gα13 and the Dbl homology domain. J Biol Chem 287 (30):25490-25500. doi:10.1074/jbc.M111.333716

31. Mamdouh Z, Kreitzer GE, Muller WA (2008) Leukocyte transmigration requires kinesin-mediated microtubule-dependent membrane trafficking from the lateral border recycling compartment. J Exp Med 205 (4):951-966. doi:10.1084/jem.20072328

32. Lovelace MD, Powter EE, Coleman PR, Zhao Y, Parker A, Chang GH, Lay AJ, Hunter J, McGrath AP, Jormakka M, Bertolino P, McCaughan G, Kavallaris M, Vadas MA, Gamble JR (2017) The RhoGAP protein ARHGAP18/SENEX localizes to microtubules and regulates their stability in endothelial cells. Mol Biol Cell 28 (8):1066-1078. doi:10.1091/mbc.E16-05-0285

33. Kaplan N, Urao N, Furuta E, Kim SJ, Razvi M, Nakamura Y, McKinney RD, Poole LB, Fukai T, Ushio-Fukai M (2011) Localized cysteine sulfenic acid formation by vascular endothelial growth factor: role in endothelial cell migration and angiogenesis. Free Radic Res 45 (10):1124-1135. doi:10.3109/10715762.2011.602073

34. Zhang J, Kim J, Alexander A, Cai S, Tripathi DN, Dere R, Tee AR, Tait-Mulder J, Di Nardo A, Han JM, Kwiatkowski E, Dunlop EA, Dodd KM, Folkerth RD, Faust PL, Kastan MB, Sahin M, Walker CL (2013) A tuberous sclerosis complex signalling node at the peroxisome regulates mTORC1 and autophagy in response to ROS. Nat Cell Biol 15 (10):1186-1196. doi:10.1038/ncb2822

35. Wessel F, Winderlich M, Holm M, Frye M, Rivera-Galdos R, Vockel M, Linnepe R, Ipe U, Stadtmann A, Zarbock A, Nottebaum AF, Vestweber D (2014) Leukocyte extravasation and vascular permeability are each controlled in vivo by different tyrosine residues of VE-cadherin. Nat Immunol 15 (3):223-230. doi:10.1038/ni.2824

36. Broermann A, Winderlich M, Block H, Frye M, Rossaint J, Zarbock A, Cagna G, Linnepe R, Schulte D, Nottebaum AF, Vestweber D (2011) Dissociation of VE-PTP from VE-cadherin is required for leukocyte extravasation and for VEGF-induced vascular permeability in vivo. J Exp Med 208 (12):2393-2401. doi:10.1084/jem.20110525

37. Schulte D, Küppers V, Dartsch N, Broermann A, Li H, Zarbock A, Kamenyeva O, Kiefer F, Khandoga A, Massberg S, Vestweber D (2011) Stabilizing the VE-cadherin-catenin complex blocks leukocyte extravasation and vascular permeability. EMBO J 30 (20):4157-4170. doi:10.1038/emboj.2011.304

38. Giannoni E, Chiarugi P (2014) Redox circuitries driving Src regulation. Antioxid Redox Signal 20 (13):2011-2025. doi:10.1089/ars.2013.5525

39. Corcoran A, Cotter TG (2013) Redox regulation of protein kinases. FEBS J 280 (9):1944-1965. doi:10.1111/febs.12224

40. Cain RJ, Vanhaesebroeck B, Ridley AJ (2012) Different PI 3-kinase inhibitors have distinct effects on endothelial permeability and leukocyte transmigration. Int J Biochem Cell Biol 44 (11):1929-1936. doi:10.1016/j.biocel.2012.07.009

41. Kwon J, Lee SR, Yang KS, Ahn Y, Kim YJ, Stadtman ER, Rhee SG (2004) Reversible oxidation and inactivation of the tumor suppressor PTEN in cells stimulated with peptide growth factors. Proc Natl Acad Sci U S A 101 (47):16419-16424. doi:10.1073/pnas.0407396101

42. Mistry JJ, Marlein CR, Moore JA, Hellmich C, Wojtowicz EE, Smith JGW, Macaulay I, Sun Y, Morfakis A, Patterson A, Horton RH, Divekar D, Morris CJ, Haestier A, Di Palma F, Beraza N, Bowles KM, Rushworth SA (2019) ROS-mediated PI3K activation drives mitochondrial transfer from stromal cells to hematopoietic stem cells in response to infection. Proc Natl Acad Sci U S A. doi:10.1073/pnas.1913278116

43. Watson RL, Buck J, Levin LR, Winger RC, Wang J, Arase H, Muller WA (2015) Endothelial CD99 signals through soluble adenylyl cyclase and PKA to regulate leukocyte transendothelial migration. J Exp Med 212 (7):1021-1041. doi:10.1084/jem.20150354

44. Corcoran A, Cotter TG (2013) FLT3-driven redox-modulation of Ezrin regulates leukaemic cell migration. Free Radic Res 47 (1):20-34. doi:10.3109/10715762.2012.733385

45. Diviani D, Osman H, Delaunay M, Kaiser S (2019) The role of A-kinase anchoring proteins in cardiac oxidative stress. Biochem Soc Trans 47 (5):1341-1353. doi:10.1042/BST20190228

46. Weber EW, Han F, Tauseef M, Birnbaumer L, Mehta D, Muller WA (2015) TRPC6 is the endothelial calcium channel that regulates leukocyte transendothelial migration during the inflammatory response. J Exp Med 212 (11):1883-1899. doi:10.1084/jem.20150353

47. Graham S, Ding M, Ding Y, Sours-Brothers S, Luchowski R, Gryczynski Z, Yorio T, Ma H, Ma R (2010) Canonical transient receptor potential 6 (TRPC6), a redox-regulated cation channel. J Biol Chem 285 (30):23466-23476. doi:10.1074/jbc.M109.093500

48. Wang Y, Ding M, Chaudhari S, Ding Y, Yuan J, Stankowska D, He S, Krishnamoorthy R, Cunningham JT, Ma R (2013) Nuclear factor κB mediates suppression of canonical transient receptor potential 6 expression by reactive oxygen species and protein kinase C in kidney cells. J Biol Chem 288 (18):12852-12865. doi:10.1074/jbc.M112.410357

49. Lohman AW, Leskov IL, Butcher JT, Johnstone SR, Stokes TA, Begandt D, DeLalio LJ, Best AK, Penuela S, Leitinger N, Ravichandran KS, Stokes KY, Isakson BE (2015) Pannexin 1 channels regulate leukocyte emigration through the venous endothelium during acute inflammation. Nat Commun 6:7965. doi:10.1038/ncomms8965

50. Lohman AW, Weaver JL, Billaud M, Sandilos JK, Griffiths R, Straub AC, Penuela S, Leitinger N, Laird DW, Bayliss DA, Isakson BE (2012) S-nitrosylation inhibits pannexin 1 channel function. J Biol Chem 287 (47):39602-39612. doi:10.1074/jbc.M112.397976
